# Simulation and identification of foodborne outbreaks in a large supermarket consumer purchase dataset

**DOI:** 10.1038/s41598-022-15584-x

**Published:** 2022-07-07

**Authors:** Peter Erdmann Dougherty, Frederik Trier Møller, Steen Ethelberg, Gunnar Øyvind Isaksson Rø, Solveig Jore

**Affiliations:** 1 Method Development and Analytics, The Norwegian Institute of Public Health (NIPH), Oslo, Norway; 2 Department of Infectious Disease Epidemiology and Prevention, Statens Serum Institut, Copenhagen, Denmark; 3 Department of Public Health, Global Health Section, University of Copenhagen, Copenhagen, Denmark; 4 Zoonotic, Water and Foodborne Infections, The Norwegian Institute for Public Health (NIPH), Oslo, Norway

**Keywords:** Epidemiology, Epidemiology, Policy and public health in microbiology, Health policy

## Abstract

Foodborne outbreaks represent a significant public health burden. Outbreak investigations are often challenging and time-consuming, and most outbreak vehicles remain unidentified. The development of alternative investigative strategies is therefore needed. Automated analysis of Consumer Purchase Data (CPD) gathered by retailers represents one such alternative strategy. CPD-aided investigations do not require trawling questionnaires to create a hypothesis and can provide analytical measures of association by direct data analysis. Here, we used anonymized CPD from 920,384 customers enrolled in Norway’s largest supermarket loyalty program to simulate foodborne outbreaks across a range of different parameters and scenarios. We then applied a logistic regression model to calculate an odds ratio for each of the different possible food vehicles. By this method, we were able to identify outbreak vehicles with a 90% accuracy within a median of 6 recorded case-patients. The outbreak vehicle identification rate declined significantly when using data from only one of two retailers involved in a simulated outbreak. Performance was also reduced in simulations that restricted analysis from product ID to the product group levels accessible by trawling questionnaires. Our results show that—assuming agreements are in place with major retailers—CPD collection and analysis can solve foodborne outbreaks originating from supermarkets both more rapidly and accurately than than questionnaire-based methods and might provide a significant enhancement to current outbreak investigation methods.

## Introduction

Foodborne diseases remain an important cause of morbidity and mortality worldwide. Some of these diseases give rise to epidemics or outbreaks, which warrant special attention. In the European Union alone, 5,175 foodborne outbreaks were reported in 2019^1^. The objectives of outbreak investigations are to stop ongoing outbreaks and to provide an evidence base for preventing future outbreaks. However, outbreak investigations are often difficult to perform successfully, particularly for outbreaks caused by contaminated foods that are widely distributed through supermarket chains.

Investigations generally consist of a two-step process, where hypotheses are first generated through descriptive analysis and interviews using trawling questionnaires, followed by analytical studies in which further interviews are conducted to prove or disprove the hypothesis. These procedures are labour-intensive, time-consuming, and prone to error^2,3^. In contrast, consumer purchase data (CPD) collected by supermarket chain databases explicitly linked to outbreak cases could provide a means to solve substantially more outbreaks and automate elements of the outbreak investigation. CPD analysis also removes the need for a classical two-step investigative process, as hypothesis generation and analytical studies are combined by direct analysis of CPD. An automated CPD analysis set-up, if introduced, thus has potential to supplement and in some situations even replace existing methods.

The viability of CPD-aided investigations is aided by the rising level of electronically recorded payments. In some areas such as the Scandinavian countries, almost all food transactions are digital^4^. These transactions may be linked to individual customers through loyalty programs or credit card data. A review from 2018 found 20 peer-reviewed papers describing CPD-assisted foodborne outbreak investigations between 2003 and October 2017^3^. In the reviewed investigations, CPD was generally used after interviews and questionnaires failed to yield sufficient hypotheses, with varying degrees of success. A search of articles published between October 2017–October 2021 using the same keywords as in^3^ yielded two additional articles detailing the use of CPD for outbreak investigation. In one, loyalty card information of a single listeriosis patient in Finland was used along with sampling of the patient’s freezer to identify the source of contaminated meatballs^5^. In the second article, CPD was used in Ireland to identify the outbreak vehicle in an outbreak of shigellosis originating from a product sold in a restaurant chain by calculating relative attack rates^6^. Of the combined 22 articles reviewed, three used CPD to generate candidate outbreak vehicles with an analytical measure of association^6–8^. Recently, we also summarized a common framework for using and reporting CPD in outbreak investigations^9^, which may aid investigators in future outbreaks.

CPD provides several potential advantages for automating outbreak investigations. If agreements are in place with database providers, CPD can in theory quickly be accessed and used to generate outbreak vehicle hypotheses. Secondly, they may be used to estimate the strength of the association between a potential vehicle and outbreak cases. However, not all personal consumption will be recorded in CPD; a sandwich bought at a food stand, dinner at a friend’s house, or purchases made by a spouse will not be recorded. Additionally, there may be barriers, legal or otherwise, that might complicate CPD usage^3^.

The work presented here was conducted to assess the feasibility of using CPD from supermarket chains to identify the vehicles of outbreaks. Here, we simulate outbreaks in a large, real-world consumer purchase dataset, and develop a model for identifying outbreak vehicles using an automated logistic regression procedure.

## Methods

### Dataset

Our CPD was obtained from *Norgesgruppen*, Norway’s largest grocery wholesaler with a 43.7% market share in 2019^10^. Norgesgruppen also runs *Trumf,* the largest customer loyalty program in Norway with almost 2.5 million registered customers^11^, recording all purchases made by Trumf-registered customers. We were generously granted access to an anonymized excerpt of CPD’s containing records of purchases of consumable items shops in 6 different municipalities in 2019. For our analyses, we partially collapsed the dataset to include unique item purchases per week per customer only, resulting in a dataset with more than 222 million recorded purchases of 30.929 items by 920.384 registered customers. The dataset does not contain batch numbers or expiry dates, as these are not included in Norgesgruppen’s records.

We were also given access to an item database with information on all listed items. Each product was categorized at two hierarchical levels of detail: primary (408 categories), and secondary (1043 categories), in addition to the actual product ID (tertiary). For example, a particular brand of blueberry would reside in the primary group “berries” and the secondary group “blueberries”.

### Implementation

Our model was implemented in the R software programming language^12^, and the statistical model built using the package glmnet^13^. All graphs were produced using package ggplot2^14^.

### Outbreak simulation

Because data were anonymized, it was not possible to perform a controlled statistical study on notified outbreaks in Norway. Instead, we simulated realistic outbreaks from the consumer purchase dataset. We first considered outbreak vehicles sold in a single supermarket chain and then expanded the model to cover two-supermarket chain-outbreaks.

To simulate outbreaks, we specified outbreak vehicles in addition to the following parameters: pathogen incubation time, delay parameter (defining the time between purchase and consumption), attack rate, background case proportion (proportion of total cases without a CPD-registered purchase of the outbreak vehicle), window size (how many days of CPD history are used for analysis dating back from case registration), outbreak start date, and outbreak duration.

**Figure 1:**
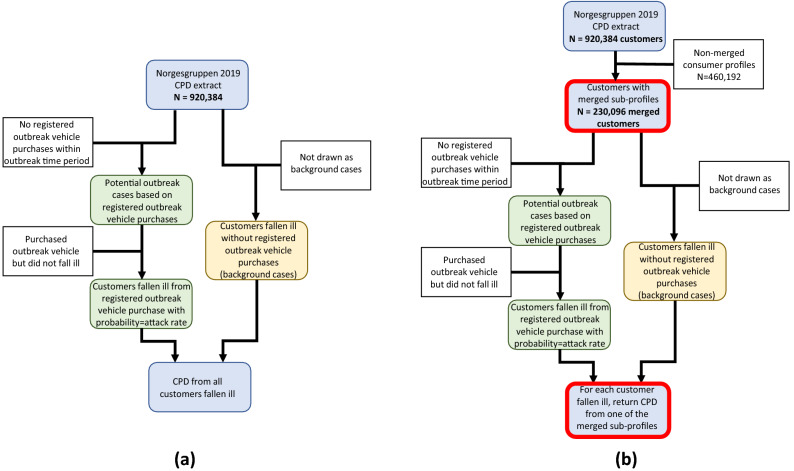
Flow charts of outbreak simulations. (**a**) Single-chain outbreak simulation. The left-hand chain in green shows how cases are generated from purchase history, while the right-hand chain in yellow illustrates how background cases are randomly selected. (**b**) Two-chain outbreak simulation. Modifications from the single-chain outbreak simulation highlighted in red.

Outbreak vehicles were randomly selected from the list of items which accumulated ≥ 500 registered purchases in 2019. This condition was applied since very rarely purchased items are highly unlikely to generate outbreaks with ≥ 5 cases. This left a pool of 12,191 possible outbreak vehicles. Flowcharts representing the outbreak simulation algorithm are shown in Fig. 1.

Two types of simulated outbreak cases were generated; cases based on purchase history, and cases based on incomplete CPD coverage (explained below). These cases generated from purchase history were termed *purchase cases*. Shown in green in Fig. 1, purchase cases were randomly drawn with probability given by the attack rate from the CPD population who purchased the outbreak vehicle within the defined outbreak timeframe. A detection date was also generated for each case, defined as the date they were registered as falling ill from the outbreak. Detection dates were generated as $purchase date + delay$, where the delay was drawn from a beta distribution $Beta\left( {\alpha = 2, \beta = 10} \right)$ and multiplied by a delay parameter *d* representing the incubation time and outbreak vehicle shelf-life. The mean value of this beta distribution is 0.167 and has a long right-hand tail, modelling that while most consumers quickly consume their purchased items, some items might be stored for a longer time. Finally, complete CPD records for each case were retrieved, dating back the number of days given by the window size parameter. Outbreak simulation is described in greater detail in the Supplementary Methods.

### Simulating incomplete CPD coverage

In any realistic outbreak scenario, some outbreak cases will not have any registered purchases of the outbreak vehicle. For this reason, a second type of cases were also randomly drawn during outbreak simulation: *background cases*. These background cases represented people who had no registered purchases of the outbreak vehicle but still fell ill from consuming it (such as by consuming the vehicle away from home).

Background cases were generated differently in two scenarios. In the first scenario, we imagine the outbreak vehicle having been sold in a single supermarket chain, while in the second scenario it would be sold in two different supermarket chains, each with their own separate CPD. In the single-chain outbreak scenario, the number of background cases was generated as a proportion of the total number of cases; if the background case proportion parameter was 0.2, then 80% of cases would be *purchase cases* and 20% will be *background cases*. Customers with fewer registered purchases were also assumed to have more unrecorded consumption, and thus an increased chance of being a background case. Hence, the chance of being randomly selected as a background case was inversely proportional to the number of weeks with registered purchases for each customer (Fig. 1a, yellow boxes).

For the two-chain outbreak scenario, we made two alterations (highlighted in red in Fig. 1b). First, we modeled customers with purchases from both supermarket chains by combining CPD profiles pairwise, where one of the sub-profiles represented CPD from supermarket chain A, and the other sub-profile represented CPD from chain B.

For this, 25% of profiles registered in the dataset were randomly selected. Next, each of these profiles were matched with a different profile in the remaining dataset with the constraint that their combined profile had registered purchases in 80–120% of weeks (weeks with purchases from both chains allow for > 100%). This ensured that profiles contain a reasonable purchase record. For example, if a customer shops 60% of weeks at the primary supermarket chain, it seems likely they might shop 20–60% of weeks at a different supermarket chain. The unmatched CPD profiles were discarded in this simulation.

After profile merging, outbreak simulation was performed as described above, generating purchase cases and background cases. However, instead of returning the complete purchase records of all case profiles, *one* of the sub-profiles was randomly selected and returned from each merged profile. This modeled a situation where we only have access to CPD from one of the two grocery chains. For comparison we ran a parallel simulation where the entire merged profile’s purchase records were returned for analysis, simulating access to CPD from both chains.

### Outbreak analysis

**Figure 2:**
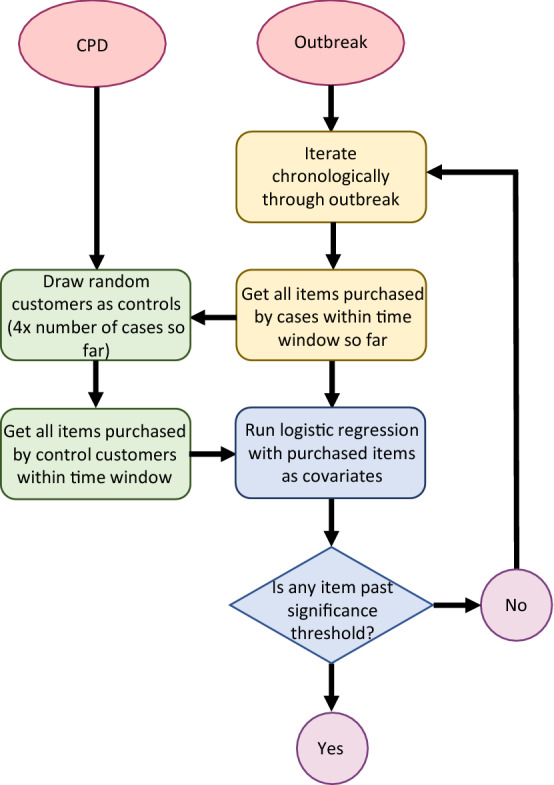
Flowchart of outbreak analysis.

After simulating outbreaks, we used logistic regression to generate odds ratios (OR) for each purchased item. The algorithm iterated chronologically through the outbreak period, analyzing newly arisen cases in order of detection date. This approach is shown in Fig. 2.

Once the number of cases exceeded five, logistic regression was initiated using item purchases as covariates. We did not attempt to solve outbreaks with less than five cases. As controls, we used the purchase histories of four randomly selected customers for each registered case. Items purchased by the recorded cases were used as regression covariates and analyzed as binomial data (1 if the covariate item was purchased by the customer, and 0 if not). Each customer’s purchases in the logistic regression were weighted by their percentage of weeks with registered purchases; again, loyal customers would be less likely to have consumed the outbreak vehicle outside of their CPD records.

This process continued either until an item reached a pre-determined OR value, the algorithm iterated through the entire outbreak period, or more than 200 cases are reached (the latter condition set due to computational limitations and only rarely encountered). In the former situation, the significantly identified item was returned as the presumed outbreak vehicle, and in the latter two situations, the algorithm returned the message that the analysis was inconclusive.

Outbreak simulation and analysis was performed across a range of important outbreak parameters; background proportion, attack rate, delay parameter (expiry date), and item purchase frequency. Each parameter was subjected to a sensitivity analysis while holding other parameters constant at default values. For each parameter value tested, 300 outbreaks were simulated using 300 randomly chosen items, and performance was quantified according to the mean outbreak vehicle ID rate and the median number of cases required to solve outbreaks.

### Analysis sensitivity to item detail level

In this section, we sought to identify the food category of outbreak vehicles based on item categories instead of item ID. One problem encountered by questionnaire-based identification methods is that they can only ask a limited number of questions, and hence operate on item categories instead of the actual consumed items. For example, the CDC national hypothesis generating questionnaire for foodborne outbreaks queries patients on their consumption of 166 different item categories such as “peperoni” and “cabbage”^15^. In contrast, our CPD-based approach has access to the specific brand of peperoni a patient purchased.

To compare our model to such a questionnaire-based approach, we used the hierarchical primary and secondary categories in addition to the actual item IDs (tertiary category). We first generated outbreaks using the single-chain outbreak simulation as before, and then ran three separate regression analyses: one with all item IDs replaced with their primary level classification (408 categories), another with secondary level classifications (1043 categories), and one with purchases classified at the tertiary level (30,929 product IDs). For the primary and secondary level classification analyses, the outbreak vehicle identification regression method returned the respective item classification level instead of product IDs.

## Results

### Description of data

A summary of the consumer purchase database used for this study is given in Table 1.

**Table 1:** Descriptive statistics of our consumer purchase dataset.

Consumer purchase dataset properties	n
Number of customers	920,384
Number of items	30,929
Total number of purchases	222,190,805
Median proportion of weeks each customer registered purchases	17%
Proportion of total purchases by top 1% of frequent customers	9%
Median number of purchases by item	176
Proportion of total purchases made up of top 1% most popular items	21%

The CPD was unevenly distributed both with respect to customer purchase frequency and item purchase frequency. The median item was purchased 176 times in 2019, and half of all customers made purchases in fewer than 10 separate weeks of 2019. Cumulative distribution plots of customers by number of weeks with registered purchases, and of items by number of purchases are provided in Supplementary Fig. S1.

### Outbreak simulation and identification

**Figure 3:**
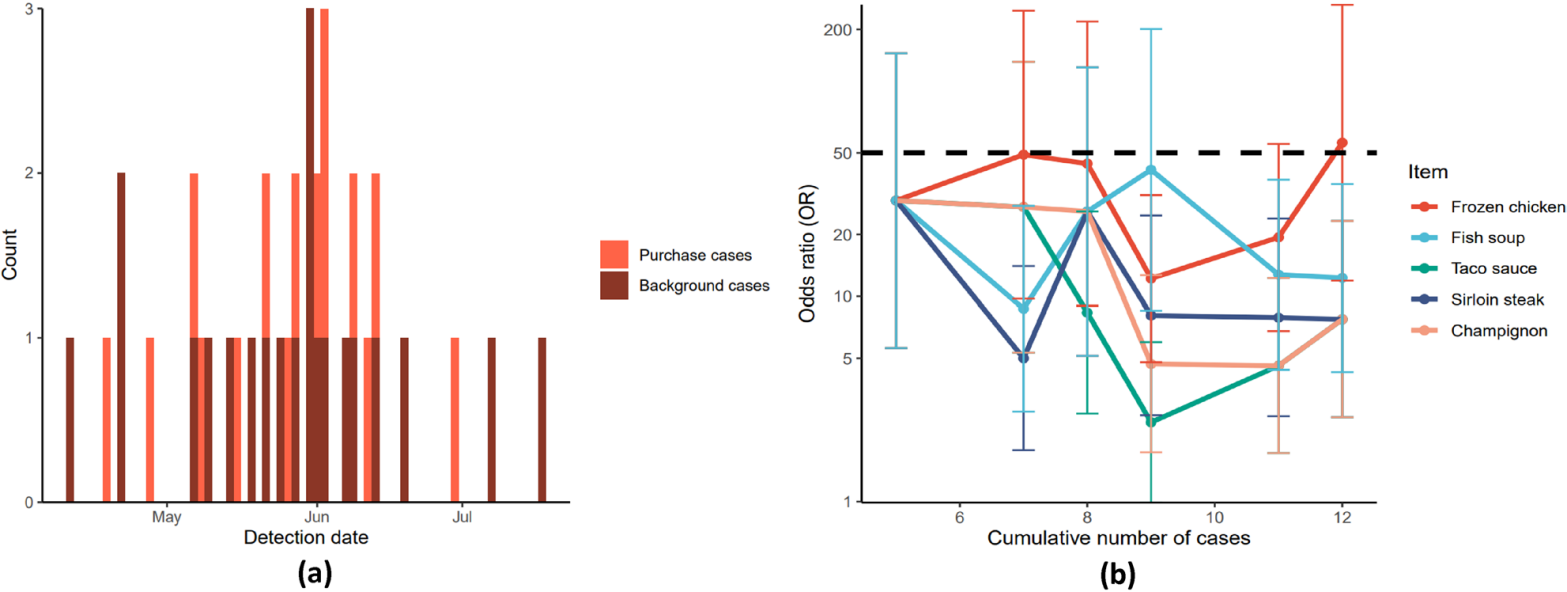
Details of a single frozen chicken outbreak and analysis. (**a**) Epidemic curve of simulated frozen chicken outbreak. Background case proportion = 0.5, delay parameter = 150 days, attack rate = 0.01, outbreak length = 50 days. N = 37 cases. (**b**) Correct identification of outbreak vehicle using logistic regression to generate odds ratios. Y-axis on log scale. After at least 5 cases are registered, the outbreak analysis algorithm of Fig. 2 is run. Every time a new case is registered, the purchase history of cases are used as covariates and case-controlled by the purchase history of randomly selected customers in the CPD. ORs are calculated for each item, and once an item reaches a pre-determined OR threshold (here 50), it is returned as the outbreak vehicle. The top 5 hits are shown in this figure.

Outbreaks were first generated according to the single-chain outbreak simulation detailed in Fig. 1a). As an example, one such outbreak where the vehicle was frozen chicken is illustrated in Fig. 3.

We then attempted to identify the outbreak vehicle using the logistic regression algorithm of Fig. 2. Outbreak vehicle identification for the chicken example is illustrated in Fig. 3b and shows the epidemic curve of the simulated outbreak along with the top 5 item hits generated.

As Fig. 3 shows, frozen chicken was successfully identified as the outbreak vehicle after 12 cases were registered, exceeding the OR threshold of 50.

### Default parameter values

Outbreaks were simulated across a range of parameters by conducting sensitivity analyses while holding other parameters constant at default values. These default parameter values are shown in Table 2 along with the ranges tested during sensitivity analysis.

**Table 2:** Summary of parameters used during outbreak simulation. Unless otherwise indicated, default values were used for simulation. Sensitivity analyses were also conducted for each parameter within the ranges shown.

Outbreak parameter	Default value	Sensitivity analysis range
Background proportion	0.2	0–0.9
Attack rate	0.01	0.001–0.1
Delay	50 days	0–300 days
Outbreak length	50 days	–
Item purchase frequency	Randomly selected	0–25th, 25th–75th, 97th percentiles

In addition to the outbreak simulation parameters, two outbreak analysis parameters were found to impact model performance: the OR threshold and the window size. These parameters were tested across a range of values to optimize performance. The effect of window size is shown in Supplementary Fig. S2 while that of the OR threshold is shown in Supplementary Fig. S3. A window size of 40 days was found to optimize the ID rate for default outbreak parameters. Although this parameter will be somewhat dependent upon the outbreak item (fresh food outbreaks may benefit from a shorter window size), this parameter seemed to be only of minor importance. Determining an optimal OR threshold is less straight-forward, as this parameter influences both the balance between correct/incorrect/no identification outcomes as well as the number of cases required for identification. From the simulations Supplementary Fig. S3, an OR threshold = 50 was chosen as an appropriate value for all further simulations.

### Impact of incomplete CPD coverage

**Figure 4:**
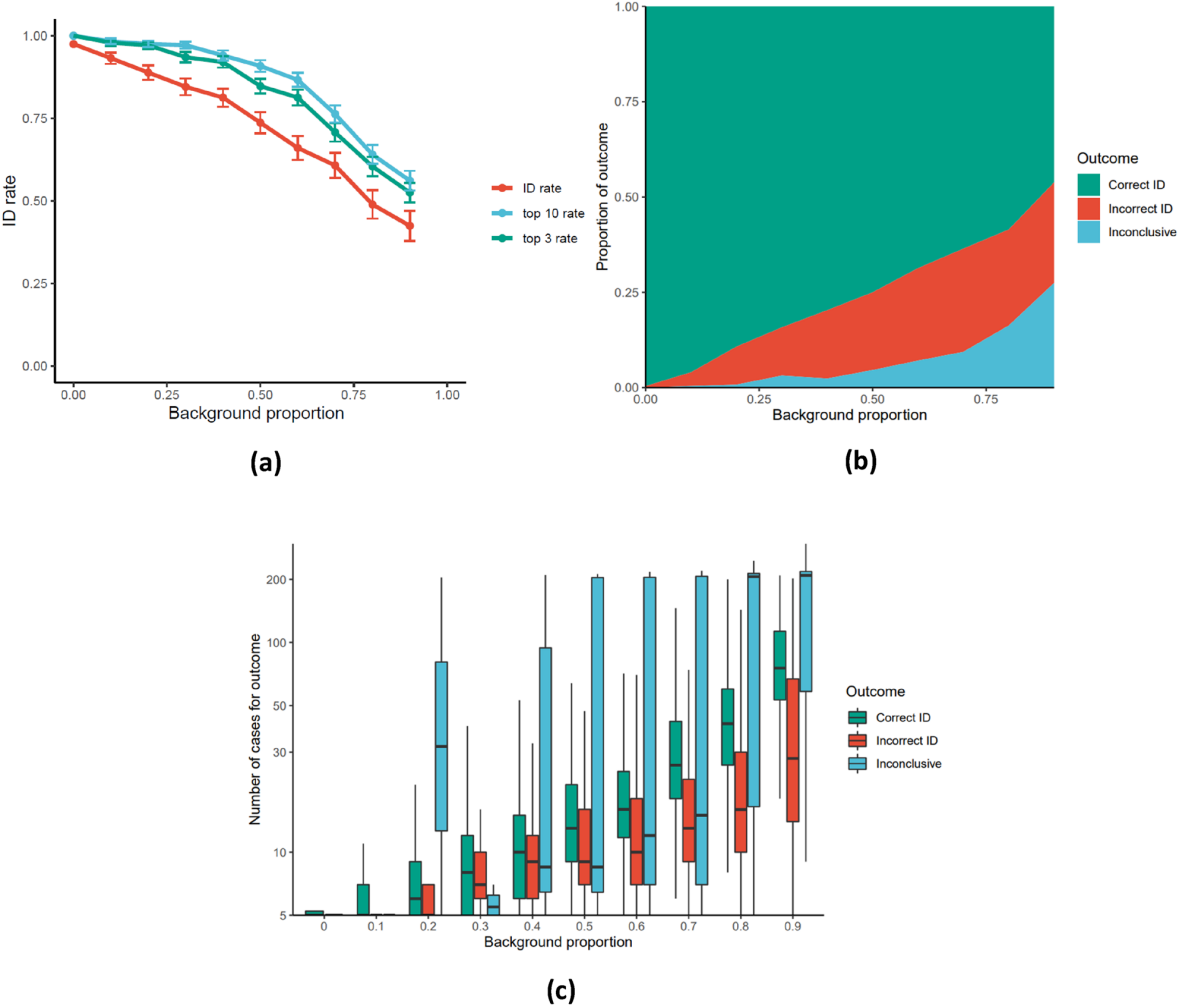
Effect of background case proportion (in range 0–0.9) on analysis of 300 simulated outbreaks with respect to (**a**) average correct identification (ID) rate top 3 rate, and top 10 rate with standard errors. (**b**) Proportional composition of each possible analysis outcome, and (**c**) boxplot of the number of cases analyzed by the model before returning the labeled outcomes. The upper and lower boundaries of the boxes indicate the group 75th (Q3) and 25th (Q1) percentile respectively, the black line within the boxes marks the group median, and whiskers extend to the least extreme data point within ± 1.5 *Interquartile range (Q3–Q1). Y-axis on log scale. Outbreak analysis was initialized at 5 cases and terminated if the number of cases exceeds 200 due to computational constraints.

Model performance was first investigated with respect to the background case proportion, representing the proportion of cases without CPD-registered purchases of the outbreak vehicle. Figure 4 shows the effect of this parameter upon outbreak vehicle identification (ID) rate, the median number of cases analyzed before the model returns a result (correct, incorrect, or inconclusive), and the relative proportions of each outcome. Figure 4a) also shows the top-3 and top-10 rates, representing the proportion of outbreaks where the true outbreak vehicle is ranked in the top 3 and top 10 list of the highest ranked outbreak vehicles respectively.

For the default outbreak parameters, Fig. 4 shows that the CPD analysis correctly identified outbreak vehicles for 90% of outbreaks after analyzing a median of 6 cases. Beyond this, we can see that model performance is highly dependent upon the background case proportion. For low background proportions, Fig. 4a) shows the ID rate approaches 100%, while in Fig. 4c) the median number of cases for algorithm termination lies close to the minimum value of five cases. However, as the background proportion increases, the ID rate decreases roughly linearly, while the median number of cases increases nonlinearly. Notably, the top-3 and top-10 rates are also significantly higher than the ID rate, highlighting the potential for generating candidate outbreak vehicles for closer investigation.

From Fig. 4b), the background proportion is also shown to impact the proportional composition of the three possible outcomes, with higher background proportions increasing both the incorrect ID rate and the inconclusive investigation rate. Interestingly, there are also considerable differences in the median number of cases analyzed for the different possible outcomes. Outbreak analyses that resulted in incorrect ID generally terminated after analyzing a lower number of cases than for analyses that resulted in correct identification. This indicates that some analyses would have been successful if the OR threshold had been higher, thus forcing the model to analyze more cases before returning an outcome.

We also ran outbreak vehicle identification on outbreaks generated by the two-chain outbreak simulation algorithm of Fig. 1b) to simulate a situation where the outbreak vehicle is sold in two different grocery chains. Both chains are assumed to have an equal market share. Two scenarios were simulated; in the first, we only have access to CPD from one of the grocery chains, while in the second we have access to CPD from both chains. Using default parameters, we found that the ID rate is severely decreased when operating on CPD from a single grocery chain vs when operating on CPD from both chains (67% vs. 92%). This result illustrates the importance of collecting CPD from all major grocery chains when the outbreak vehicle is sold in several chains.

### Outbreak product group identification

**Figure 5:**
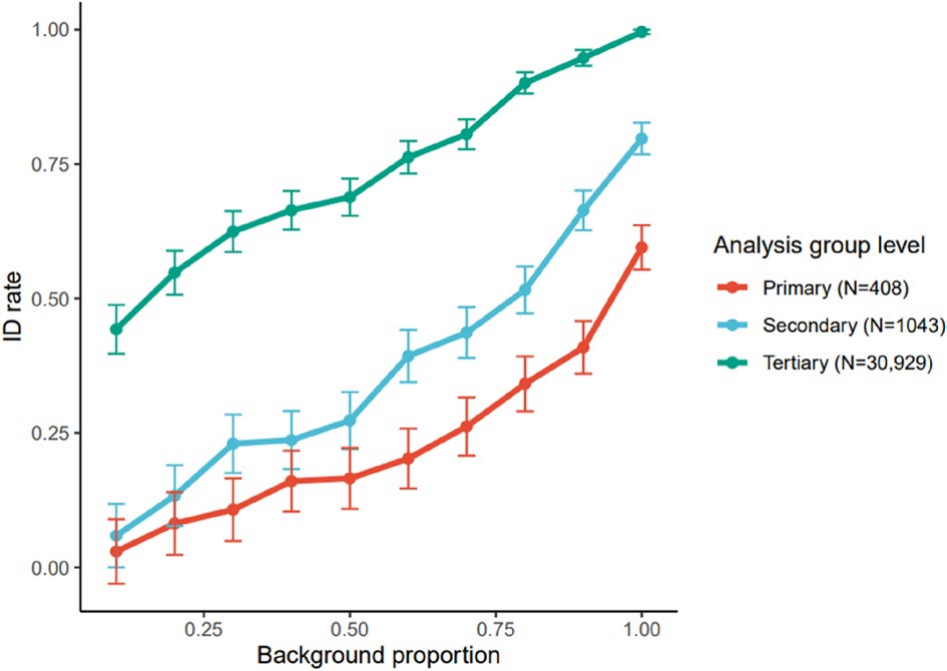
Average ID rate against background proportion for analysis using primary, secondary, and tertiary (product ID number) product groups with standard errors. 300 outbreaks for each data point are simulated as before, but instead of only using the product ID number as covariates, simulations were also run using the primary and secondary product groups for each registered purchase. The identification algorithm then attempted to return the relevant product group.

Outbreak vehicle identification was attempted using the product group categories previously described. This was done to imitate the item categories used by outbreak investigations which use case interviews and questionnaires. When using the primary and secondary product groups, the outbreak vehicle identification algorithm attempts to return the relevant product group category. The results of these simulations are shown in Fig. 5.

Figure 5 shows that using primary (34% ID rate) and secondary (52% ID rate) product groups resulted in drastically lower performance relative to using tertiary (90% ID rate) product ID numbers, analogous to questionnaire-assisted investigations.

### Sensitivity analyses of attach rate, delay, and item purchase frequency

Next, the impact of attack rate and the delay parameter were investigated with respect to ID rate. Interestingly, these simulations showed that ID rate is only slightly impacted by either of these parameters (less than ± 10% ID rate within the ranges tested). This data is included in Supplementary Fig. S4.

Finally, the purchase frequency of outbreak vehicles was considered. After ranking all items by the number of times they were purchased, outbreak vehicles were drawn from three different sets of items: low (0-25th percentile), mid (25th–75th percentile), and very high frequency (97th–100th percentile) items. Using the default outbreak parameters, three sets of simulations were run. Here, a more significant difference was found between groups: the ID rates for the low, medium, and very high frequency groups was 87%, 93%, and 79%, respectively. This data is also shown in Supplementary Fig. S5.

## Discussion

With this study, we sought to use real-life CPD collected by supermarket chains to identify outbreak vehicles in simulated foodborne outbreaks. Using a wide range of outbreak parameters and vehicles, we found that the most important parameter for the ability to detect outbreaks using CPD is the completeness of CPD coverage, quantified by the background case proportion. For simulated outbreaks with a background case proportion of 20%, our model was able to correctly identify outbreak vehicles in a median of 6 cases with a 90% correct ID rate. This shows that most outbreaks are solved almost immediately upon initializing analysis at five cases. The model also identified shortlists of the top 3 and top 10 most likely outbreak vehicles with an even higher rate of accuracy. These shortlists could then be subjected to more intensive investigation in a real outbreak scenario. Generating such shortlists may be particularly of interest if investigating outbreaks with fewer than the minimum five cases considered here, where a definitive outbreak vehicle is unlikely to be found.

To compare this CPD-based approach to more traditional questionnaire-based investigations, we analyzed outbreaks using the product group categories obtainable by questionnaires instead of exact item IDs. The number of categories in the primary (N = 408) and secondary (N = 1043) level product groups was quite generous; with traditional outbreak investigation, it is simply not realistic for people to remember and report their consumption of this many categories. For comparison, the CDC national hypothesis generating questionnaire queries people on their consumption of 166 item groups, although in contrast to the item groups specified in our dataset, the CDC’s item groups have been chosen to optimize outbreak investigations with as few questions as possible.

Even with a relatively high number of product categories, this analysis resulted in a huge drop in model performance (34% and 52% ID rate for primary and secondary categories respectively). Interestingly, these ID rates are also comparable to real-world results; according to the Center for Science in the public interest, 39% of foodborne outbreaks reported to the CDC in 2013 were solved^16^. Although our simulation certainly does not capture all aspects of a questionnaire-based approach, it does demonstrate that identification at product group levels is very inefficient compared to at the item ID level, and that CPD-based investigations have the potential to greatly improve upon existing foodborne investigative techniques.

To better test simulation parameters in our analysis model, we conducted several sensitivity analyses. The most impactful outbreak parameter was clearly the background proportion, with both the incorrect ID rate and the inconclusive rate greatly increased at higher background proportions. The incorrect ID rate is particularly important to bear in mind, given the economic cost of product recall. This is not a risk unique to CPD-analyses, as epidemiological studies can only ever give statistical association. Just as with standard questionnaires, further investigation such as microbiological product testing would often be necessary for conclusive proof of outbreak vehicle.

With increasing background proportion, the number of cases required for the outbreak analysis algorithm to terminate also increases. Interestingly, the median number of cases for analyses that resulted in false ID was always lower than for analyses that resulted in correct ID, suggesting that some unsuccessful analyses would have been successful if more cases had been analysed. However, sensitivity analysis shows that increasing the OR threshold also increases the number of cases required for identification. In an outbreak scenario, it may be useful to generate shortlists of likely outbreak vehicles early in an outbreak for further investigation, while keeping in mind that more data may change the analysis results.

Diving deeper into the differences between outbreak vehicles revealed that very high frequency items (97th purchase frequency percentile) generated outbreaks significantly more difficult to solve than mid frequency items (25th–75th percentile). Since certain staple goods are so highly purchased, they are likely to be in the purchase history of control cases by chance without causing the control cases to fall ill. This can be very impactful; the most purchased item in the CPD (a brand of banana) constitutes 0.8% of all purchases.

Interestingly, the attack rate and delay parameter were not found to greatly impact model performance. Although initially surprising, the insignificance of the attack rate does make sense. Even if 99.9% of customers who purchased the outbreak vehicle do not fall ill, 80% of cases (at the default background proportion) will still have registered purchases of the outbreak vehicle. Although very low attack rates generate smaller outbreaks which may be more difficult to solve, this was not a significant effect in our model. Similarly, long delay parameters will only reduce model performance if the number of days between a customer’s outbreak vehicle purchase and falling ill exceeds that of the analysis window size (40 days). Since most outbreaks are solved quickly, this rarely came into play.

In addition to high background proportions and high purchase frequencies, our model also struggled in scenarios where the outbreak vehicle is sold in two grocery chains, but we only have access to CPD from one of the chains (67% ID rate vs 92% with access to CPD from both). The reduced performance is not especially surprising; this situation is comparable to the single-chain outbreak simulation with a high background case proportion. Clearly, a CPD-based outbreak investigation benefits from a pre-existing relationship with major grocers. With the permission of the patients, their various CPD-records could be merged to a single profile containing all their recorded purchases.

More generally, poor coverage is likely to be a limitation of CPD-based investigations. For this reason, CPD approaches are particularly well-suited in countries with high usage of electronic payments, an oligopolistic grocery market, and a low frequency of eating out. These conditions are certainly met in Norway; less than 4% of transactions in Norway are by cash^17^, Norgesgruppen has a 44% market share^10^, and most Norwegians report eating out less than once a month^18^. Despite this, in our CPD the median proportion of weeks in which customers’ recorded purchases was 17%, implying coverage of total consumption was quite low. One possible explanation might be households with multiple consumers registered to different loyalty accounts. If a two-person household alternates shopping evenly so that each person only shops every other week, both will have “low” coverage even if they only eat food from Norgesgruppen. These profiles should also be merged in a real outbreak scenario, using their shared address to form a single household profile.

Although our CPD-based approach performed very well on the simulated outbreaks, it is not easy to ascertain how well these simulations model real-world outbreaks. There are relatively few^3,6^ published studies demonstrating the usage of CPD for foodborne outbreaks and it is therefore important to test the model on real-world outbreak data. Recently, we initiated a pilot study for the collection of CPD from volunteers, aiming to eventually investigate real-world outbreaks with a CPD-based approach^19^. With this, we imagine a future with private–public partnerships allowing streamlined data collection for outbreak investigations with consumer consent. This study also shows that the concerns raised regarding legal barriers to CPD-collection^3^ are surmountable.

In conclusion, our simulations showed that access to CPD from a large supermarket chain provides the potential to solve outbreaks quickly and accurately. By combining hypothesis generation and analytical study into a single step to generate quantitative measures of association, CPD analysis represents a major simplification of the traditional investigative process. However, limitations of this approach include low market coverage and highly purchased outbreak vehicles. Due to this, CPD investigations would seem particularly well-suited for countries with a high saturation of electronic payments and an oligopolistic grocery market. In scenarios where such conditions are not completely met, CPD may still provide an important supplement to outbreak investigations conducted using traditional questionnaires and case interviews. If implemented, CPD would offer foodborne outbreak investigators a very powerful tool which might help reduce the public health burden of foodborne illnesses.

## Supplementary Information

Supplementary Information.

## Data Availability

The data that support the findings of this study are available from Norgesgruppen but restrictions apply to the availability of these data, which were used under license for the current study, and so are not publicly available. Data are however available from the authors upon reasonable request and with permission of Norgesgruppen.
