# Supplementary material for: Simulation and identification of foodborne outbreaks in a large supermarket consumer purchase dataset
Source: Sci Rep. 2022 Jul 7;12:11491. doi: 10.1038/s41598-022-15584-x (PMC9263146; doi:10.1038/s41598-022-15584-x)
Supplement: Supplementary file 1 — Supplementary Information. [file 41598_2022_15584_MOESM1_ESM.docx]

**Simulation and identification of foodborne outbreaks in a large supermarket consumer purchase dataset**

Peter Erdmann Dougherty^1^, Frederik Trier Møller^2^, Steen Ethelberg^2,3^, Gunnar Øyvind Isaksson Rø^1^, Solveig Jore^4*^

^1^ Method Development and Analytics, The Norwegian Institute of Public Health (NIPH), Oslo, Norway

^2^ Department of Infectious Disease Epidemiology & Prevention, Statens Serum Institut, Copenhagen, Denmark

^3^ Department of Public Health, Global Health Section, University of Copenhagen, Copenhagen, Denmark

^4^ Zoonotic & Foodborne Infections, The Norwegian Institute for Public Health (NIPH), Oslo, Norway

^*^ Corresponding author: solveig.jore@fhi.no

**Supplementary Methods**

Upon selecting outbreak parameters, two types of cases are generated. First, cases are generated based on purchase history. These are termed *purchase cases*. All customers who purchased the outbreak vehicle between $[start date, start date+ outbreak length]$ are selected. Each of these customers are assigned as cases with probability given by the attack rate. A detection date is also generated for each case, defined as the date they are registered as falling ill from the outbreak. Detection dates are generated as $purchase date+delay$, where *delay* is the elapsed time from customer purchase of the outbreak vehicle to registration

$delay= T_{storage}+T_{incubation}+ T_{registration}$ (1)

In equation (1), *delay* is shown as the sum of; $T_{storage}$, the elapsed time from purchase date to outbreak vehicle consumption, $T_{incubation}$, the elapsed time from outbreak vehicle consumption to symptom onset, and $T_{registration}$ is the elapsed time from symptom onset to registration of the case for outbreak investigation. Of the three, $T_{storage}$ is assumed to be the most significant, as non-perishable goods can lie in the cupboard for months before being eaten. To reduce the number of variables, $delay$ was treated as a single variable.

Not all customers in each outbreak will have the same delay between purchase and registration. Some consumers will consume the outbreak vehicle immediately upon purchase, while others may wait days or even months. To model this, we generated delays for each sickened consumer within an outbreak with $delay=d*Beta(\alpha=2, \beta=10)$, where *d* is the delay parameter and Beta is the beta distribution. Delay parameters between 0 and 300 days were tested, and the default value for simulations was 50 days. The beta distribution with parameters $\alpha=2, \beta=10$ was chosen as it models that most items are consumed soon after purchase, but has a long tail, modelling that while most consumers quickly consume their purchased items, some items are stored for a long time.

In addition to purchase cases, background cases are also drawn. Since all cases are assumed to stem from a specific outbreak, background cases represent people who had no registered purchases of the outbreak vehicle but still fell ill from consuming it, for example at a friend’s house. Hence, the number of background cases is generated as a proportion of the total number of cases; if the background case proportion is 0.2, then 80% of cases will be purchase cases and 20% will be background cases.

To generate background cases, a background case rate is calculated for the entire CPD population based on the background case proportion number of purchase cases as

$p_{background}(prop) =N_{purchase cases}*(\frac{1-prop}{prop})/N_{CPD}$ (2)

Where $p_{background}$ is the background rate, $N_{purchase cases}$ is the total number of purchase cases generated in this outbreak, $prop$ is the set background proportion, and $N_{CPD}$ is the number of customers in the CPD. Customers from the entire CPD are then drawn as background cases with a binomial distribution parameterized by $p_{background}$. This binomial draw was weighted so that each customer’s probability of being drawn is inversely proportional to their number of weeks with registered purchases in the dataset; customers with fewer registered purchases are assumed to have more unrecorded consumption, and thus an increased chance of being a background case.

After this, each background case is assigned a detection date as before, and their purchase history between $[start date, detection date]$ is retrieved. Finally, the background cases and the purchase cases are merged, and returned as the complete outbreak. The two types of cases are then indistinguishable.

**Supplementary Figure S1**

**(b)**

**(a)**


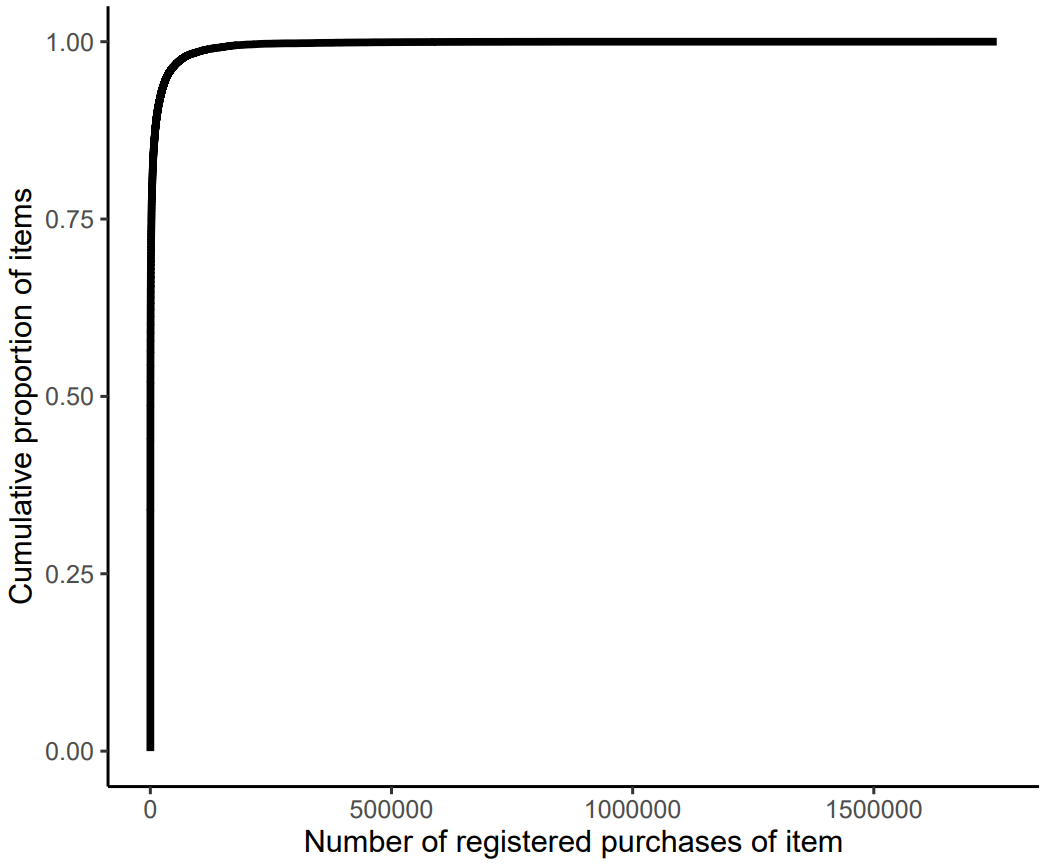

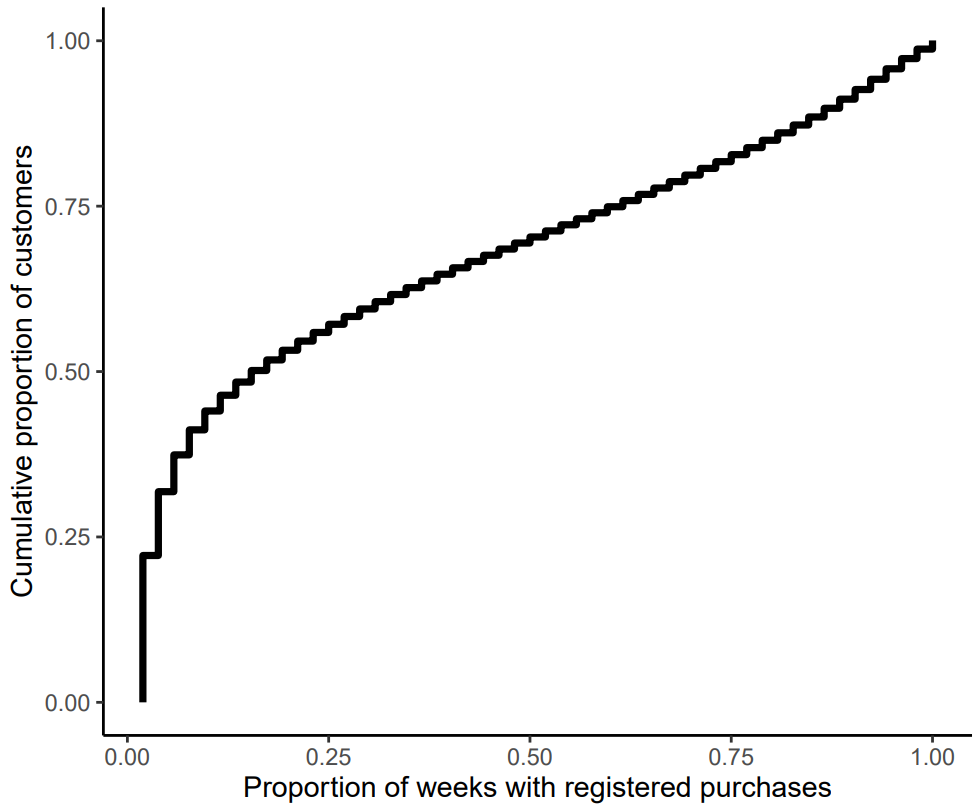


**Fig. S1:** Cumulative distribution plots of **a)** the 30,929 items for sale vs their number of registered purchases and **b)** the 920,834 customers in the CPD vs their number of weeks in 2019 with registered purchases.

**Supplementary Figure S2**

**
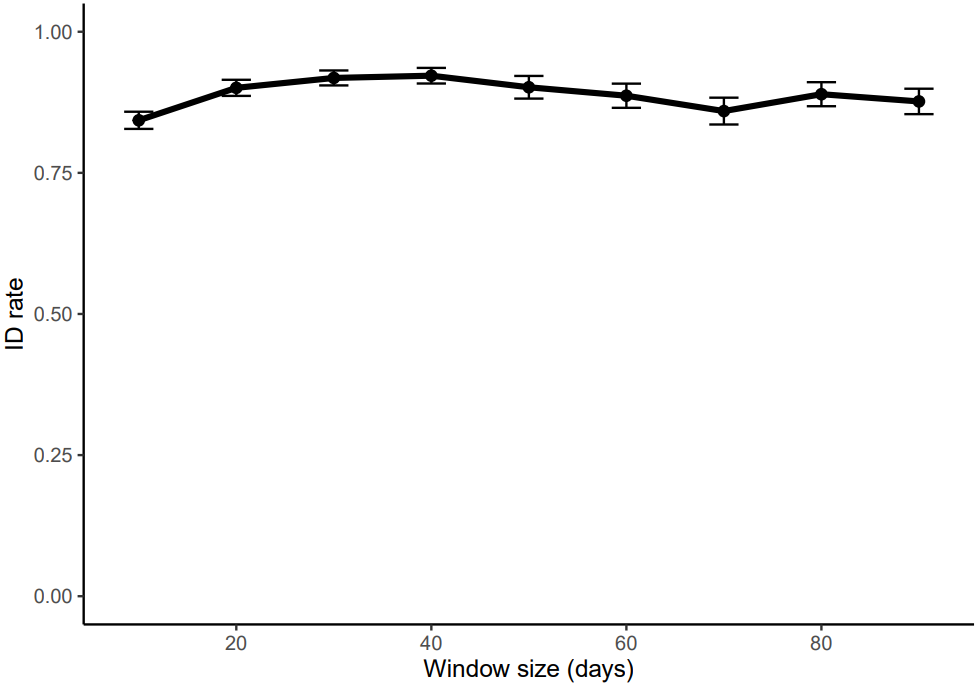
**

**Fig. S2**. Average ID rate plotted against window size with standard error. Window size is the number of days of CPD history used during analysis, dating back from case registration. The average ID rate peaks (0.92) at window size = 40 days, and this value is used for all other simulations. Analysis performed on 300 outbreaks per data point.

**Supplementary Figure S3**

**(b)**

**(a))**


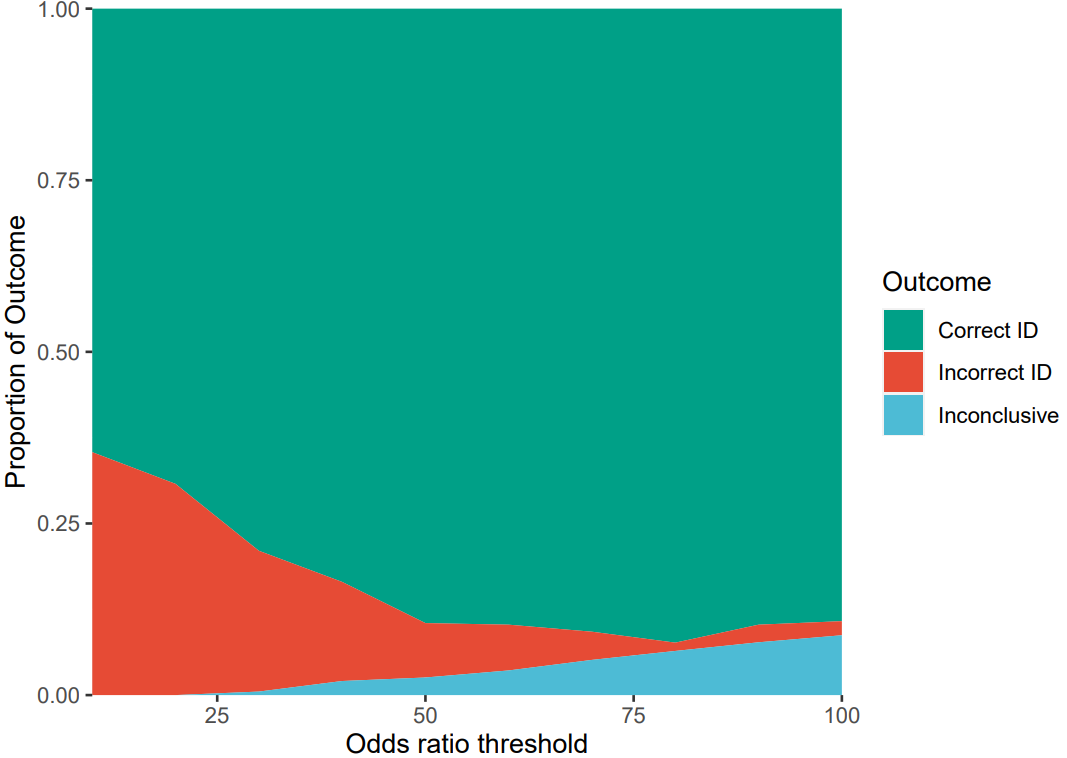

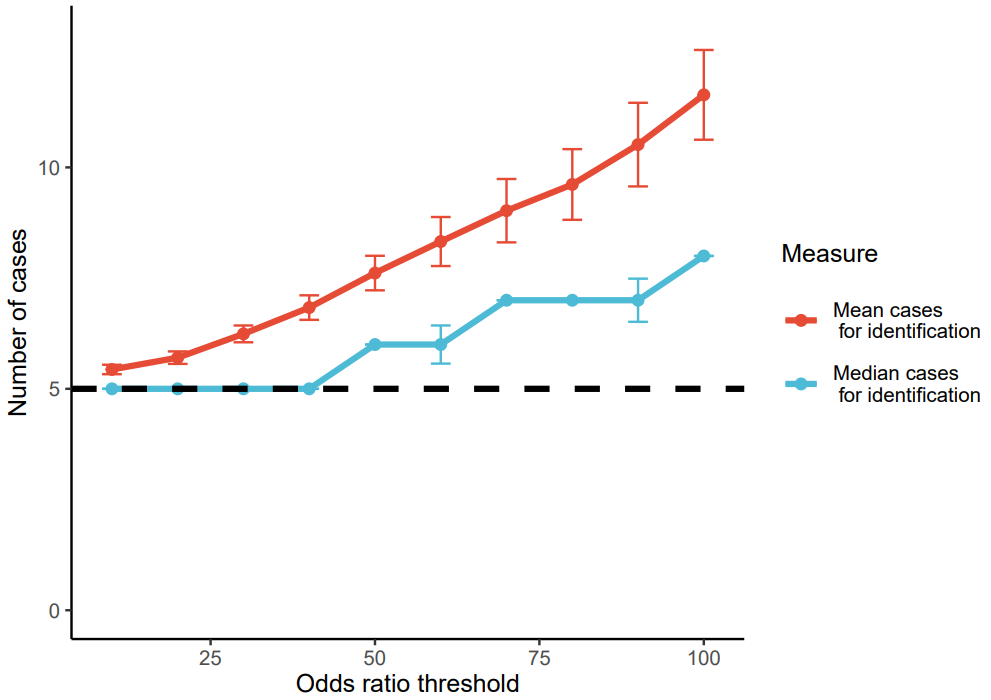


**Fig. S3.** Effect of the odds ratio threshold on **a)** the relative proportions of the possible outcomes (correct ID, incorrect ID, and inconclusive analysis), and **b)** mean and median number of cases with standard errors required by the model to identify the correct outbreak vehicle ID. Outbreak analysis is initialized at 5 cases and terminated if the cumulative number of cases exceeds 200. From these figures, an OR threshold = 50 was chosen. Although the incorrect ID rate decreases from 8% to 1% if the OR threshold is increased further to 80, this comes at the cost of an increased number of cases required to solve outbreaks (the median number of cases rises from 6 to 7 while the mean rises more). Additionally, this decreased incorrect ID rate only results in a 2% increased correct ID rate, as the percentage of inconclusive investigations is instead increased by 6%. For these reasons, an OR threshold = 50 was decided upon as an acceptable compromise between efficiency and accuracy.

**Supplementary Figure S4**


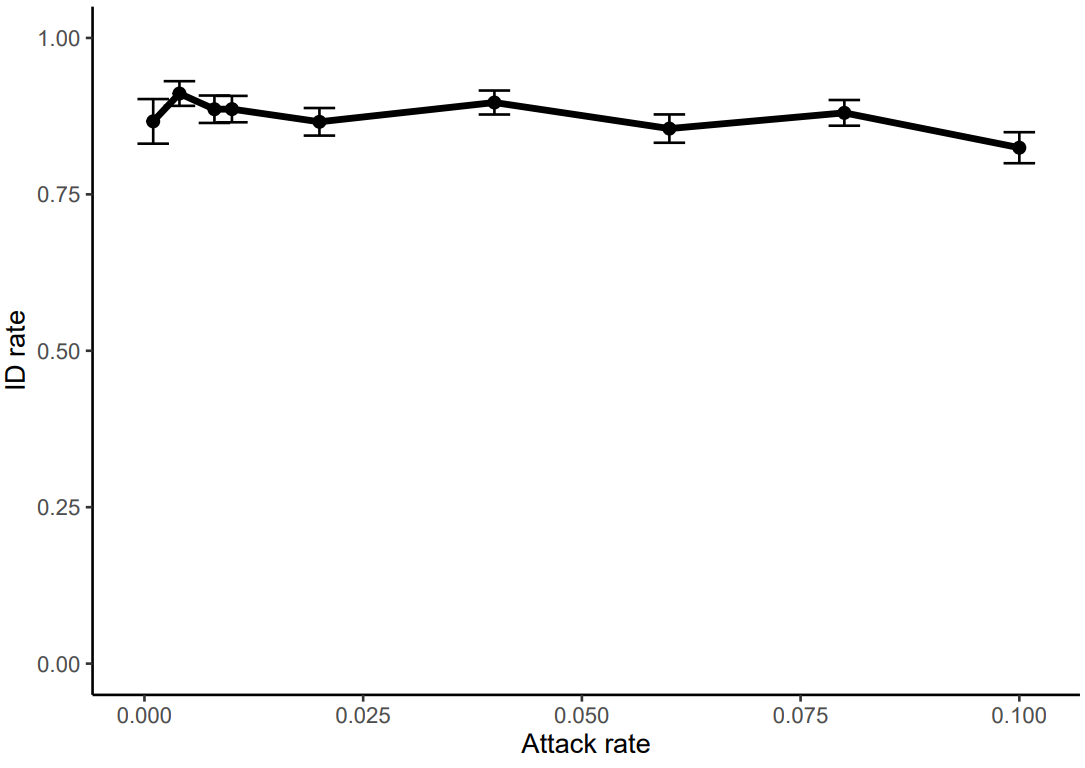

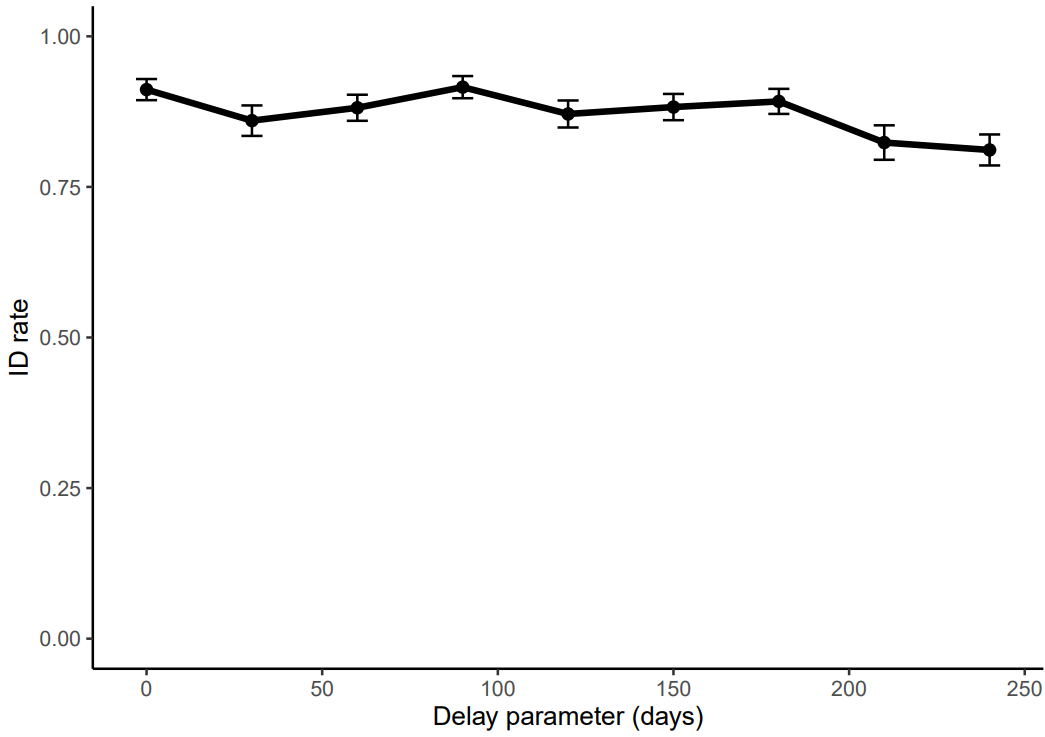


**Fig. S4:** Average ID rate with standard error against **a)** Attack rate and **b)** Delay parameter. Analyses performed on 300 outbreaks per data point.

**Supplementary Figure S5**

**
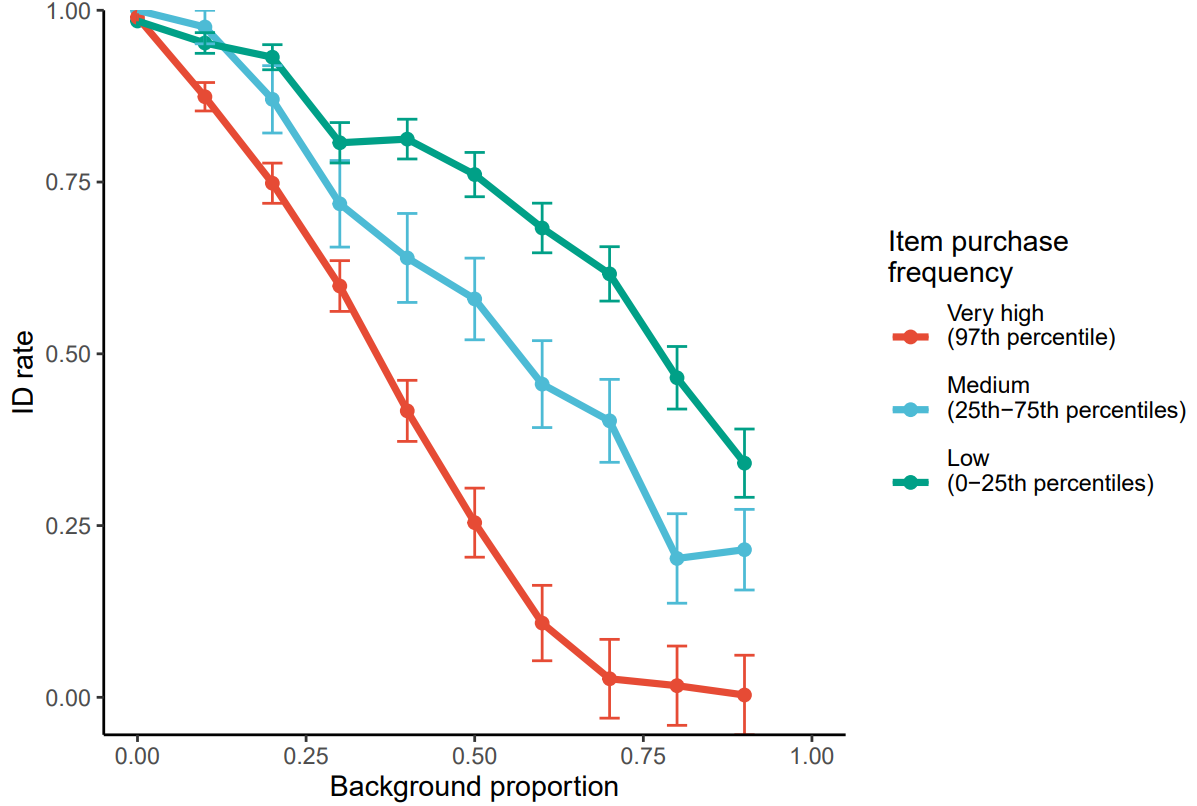
**

**Fig. S5:** ID rate against background case proportion for different outbreak vehicle purchase frequencies groups.
